# The Economics of Group Antenatal Care: A Systematic Review and Narrative Analysis

**DOI:** 10.1007/s10995-026-04276-x

**Published:** 2026-05-13

**Authors:** M. Elske van den Akker-van Marle, Nathalie Leister, Ashna D. Hindori-Mohangoo, Ilir Hoxha, Marlies E. Rijnders, Christine McCourt

**Affiliations:** 1 Department of Biomedical Data Sciences, Leiden University Medical Center, Leiden, The Netherlands; 2 School of Health & Medical Sciences, Centre for Maternal &Child Health Research, City St George’s, University of Londen, London, UK; 3 Department of Maternal, Infant and Psychiatric Nursing, School of Nursing, University of São Paulo, São Paulo, Brazil; 4 Foundation for Perinatal Interventions and Research in Suriname (Perisur), Paramaribo, Suriname; 5 Action for Mother and Children, Prishtina, Kosovo; 6 Evidence Synthesis Group, Prishtina, Kosovo; 7 The Dartmouth Institute for Health Policy and Clinical Practice, Geisel School of Medicine at Dartmouth, Lebanon, USA; 8 The Netherlands Organization for Applied Scientific Research, Leiden, The Netherlands

**Keywords:** Keywords, Group care, Antenatal care, Pregnancy, Economics, Costs

## Abstract

**Introduction:**

Group antenatal care (GANC) is an alternative to traditional individual antenatal care (IANC), which combines health assessment, interactive learning, and community building in group sessions. GANC has been associated with positive health outcomes. To scale up GANC, more evidence is needed on the financial implications of its initial implementation and (long-term) cost-effectiveness. This study aims to review and synthesise the available evidence on the economics of GANC.

**Methods:**

We searched for observational and experimental studies assessing the cost aspects of implementing and running GANC with or without comparison with IANC.

**Information sources:**

We searched PubMed, EMBASE, and Ovid Emcare up to 22 August 2024 using keywords and controlled vocabulary without restriction by year of publication.

**Quality Assessment:**

CASP Economic Evaluation Checklist.

**Data Synthesis:**

Narrative synthesis.

**Results:**

A limited number of studies (n = 9) addressing the costs and/or benefits of GANC were eligible to be included in the review. These studies varied considerably in setting, design, quality, type of cost data, cost categories included and perspective used.

**Conclusion:**

Evidence on the costs of GANC is sparse. Future studies of the lifetime costs and health outcomes of GANC compared with IANC are needed to gain insight into the cost implications and cost-effectiveness of GANC and to scale up its implementation.

**Trial Registration:**

PROSPERO 2023 CRD42023454379.

**Supplementary Information:**

The online version contains supplementary material available at 10.1007/s10995-026-04276-x.

## Significance

What is Already Known on this Subject?

What this Study Adds?

Group antenatal care (GANC) has been associated with positive outcomes, including reduced rates of preterm birth and low birth weight, improved maternal satisfaction, health behaviour and ANC attendance. Better outcomes of GANC were also found among women in vulnerable situations. To scale up GANC, more evidence is needed on the financial implications of its initial implementation and (long-term) cost-effectiveness.

This study summarises the currently available knowledge, which appears to be sparse. Gaps in knowledge are identified and guidance for future research is provided.

**Supplementary Information:**

The online version contains supplementary material available at 10.1007/s10995-026-04276-x.

## Introduction

Group antenatal care (GANC) is a healthcare model that aims to provide a more client-centred and holistic approach by replacing traditional one-to-one antenatal visits, also referred to as individual antenatal care (IANC), with interactive group sessions, allowing women to engage in discussions, share experiences, and receive comprehensive health assessments, fostering a supportive environment for expectant mothers and parents (Sadiku et al., 2023). In some settings also group postnatal care (GPNC) or an integrated antenatal and postnatal model has been implemented.

Most GANC models are based on the CenteringPregnancy® model, with groups of 6–12 women/parents meeting approximately 8–10 times (generally following the usual antenatal care schedule for the context) in two-hour sessions during pregnancy and usually including one group postnatal reunion visit. These sessions are guided by two facilitators, with at least one of them a licensed healthcare provider, such as a midwife (Rising, 1998). The health assessments are advised to be brief and to take place in the group space, with women encouraged to participate in self-checking for routine health checks such as blood pressure.

GANC has been associated with positive outcomes, including reduced rates of preterm birth (Ickovics et al., 2007, Pickleseimer et al. 2012) and low birth weight (Carter et al., 2016), improved maternal satisfaction (Sadiku et al. 2023), health behaviour (Wagijo et al., 2023) and ANC attendance (Grenier et al., 2019, Lori et al. 2024). Better outcomes of GANC were also found among women in vulnerable situations (Byerly & Haas 2017, Crockett et al. 2022). Worldwide, the increased implementation efforts of GANC are seen in various high, middle, and low-income countries (Martens et al., 2022).

However, despite this wider implementation and the promising association with improved outcomes, sustainability and widespread implementation of GANC face several challenges. These include logistical hurdles such as securing appropriate space for group sessions, training healthcare providers to facilitate GANC, ensuring continuity of facilitators and participation from expectant mothers, and also the lack of knowledge on the financial implications of its initial implementation and cost-effectiveness in the long run. Uncertainty about the initial costs associated with implementing GANC, and the subsequent costs of sustaining this care approach, especially in relation to unknown (future) financial benefits, may deter healthcare facilities or policymakers from adopting this model.

Hence, in this review, we aim to provide a comprehensive overview of the current available knowledge and evidence on the economics of GANC, identify remaining knowledge gaps, and guide future research.

## Methods

### Search Strategy and Data Sources

With the help of an experienced librarian, we designed and ran a systematic search strategy. This strategy involved searching multiple databases until 22 August 2024. The databases searched were PubMed, EMBASE and Ovid Emcare. The search strategy used keywords supplemented with controlled search terms on economics, group and individual care, and antenatal care (see Supplementary Table 1).

### Study Selection

All retrieved citations were imported into one Endnote library in order to delete duplicates. Citations remaining after this step were exported into the bibliographic reference management software Rayyan for eligibility screening. Two reviewers (NL and MA) worked independently to assess study eligibility in two phases (title and abstracts followed by full-text assessments). In case of disagreement between both reviewers, a third reviewer (CM) was consulted.

Eligible studies reported primary data on any cost outcomes of implementing or running a model of ANC, or ANC and postnatal care that include participants meeting in a group (at least 4 women), this includes also comparisons between the costs of GANC and IANC. Only articles in the English language were included. Conference abstracts were excluded. Furthermore, we excluded studies in which groups were provided outside mainstream health care (e.g. by charity groups), one-off groups, groups not including clinical care (such as classes only) and groups not involving any health professional input (such as peer-led groups) (see Table 1).

**Table 1:** Inclusion and exclusion criteria for the review

	Inclusion	Exclusion
Group size	At least 4 people	Less than 4 people
Exposure	GANC, or GANC and GPNC as part of the regular care and involving clinical care by a healthcare professional	Individual care, group care provided outside the mainstream health care, one-off groups, groups with no clinical care, groups without health professional input
Outcomes	Primary data on any cost outcomes	With no cost outcomes
Language	English	Any other language
Time period	No restriction	
Publication type	Peer review articles	Conference abstracts

*GANC* group antenatal care, *GPNC* group postnatal care

### Data Extraction and Study Quality Assessment

Details of the studies, including study setting, study design, participants, interventions, methodological and economic aspects and results, were extracted by the principal investigator and checked by the other reviewers using a standardised form developed for this review. Cost items were converted from their original year and currency to US dollars, price level 2022 using World Bank data on consumer price index and exchange rate (World Bank 2024a, 2024b).

Pairs of authors independently performed a critical appraisal of the studies using the CASP Economic Evaluation Checklist (CASP, 2023). This checklist was chosen even though not all the studies were designed as economic evaluations. We chose one form, particularly this one because we wanted to compare the studies from an economic perspective, which is not covered in standard checklists for trials and cohort studies. If a particular item in this economic evaluation checklist did not apply to the study in question, it was left blank. Also, quality issues not related to or affecting the economic perspective or analysis were left out of consideration.

### Data Synthesis

The paucity and heterogeneity of included studies were anticipated to preclude a quantitative synthesis. Therefore, we intended to summarise the evidence narratively by summarizing cost estimates of implementing and running GANC (Campbell et al., 2020; McKenzie & Brennan 2019) and where possible compared to IANC. To enable comparisons of cost estimates, costs have been converted to the same price level (year 2021) using consumer price indices and healthcare purchasing power parities (PPP) were used to convert expenditure into a common unit (World Bank 2024a, 2024b). In this way, comparison of expenditure between countries only reflects differences in the volume of goods and services consumed.

Owing to the nature of the study, no ethical approval or patient consent is applicable. The study was registered in PROSPERO with registration number CRD42023454379.

## Results

**Fig. 1:**
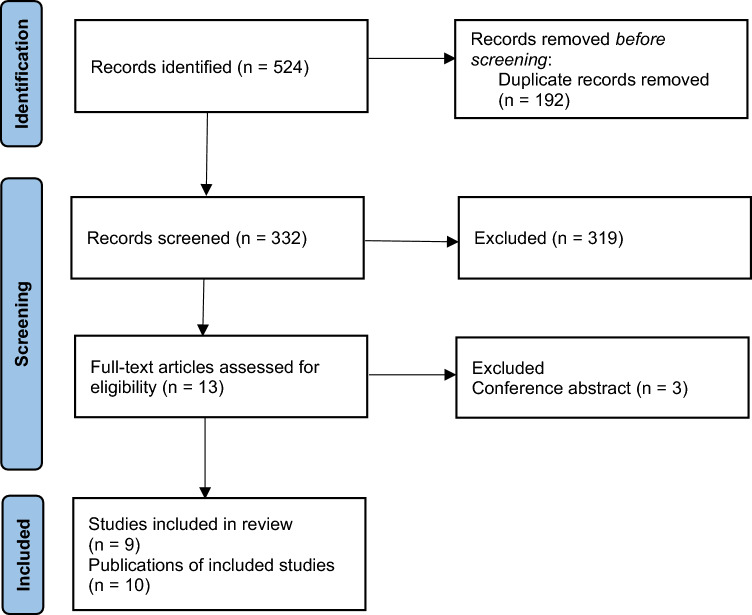
Flow diagram of publication selection. From: Page MJ, McKenzie JE, Bossuyt PM, Boutron I, Hoffmann TC, Mulrow CD, et al. The PRISMA 2020. statement: an updated guideline for reporting systematic reviews. BMJ 2021;372:n71. https://doi.org/10.1136/bmj.n71

Figure 1 describes the flow of studies through our search and selection process. We identified 524 records, after removing duplicates, we screened 332 titles and abstracts, excluding 319 records mainly because they did not report on group care or costs. After reading the full text of 13 articles, 9 eligible studies remained, reported in 10 publications (for one study we identified both a full study report and a journal article). Table 2 describes the study characteristics. Only four studies were conducted outside the USA (Barnes et al., 2017a, 2017b (UK), Harsha Bangura et al., 2020 (Nepal), McKinnon et al., 2020 (Senegal), Jans et al., 2023 (Netherlands)), of which two in a low- or middle-income country (LMIC) settings (Harsha Bangura et al., 2020; McKinnon et al., 2020). In these LMICs the number of group sessions is lower than in other countries (5—6 compared to 8—14 GANC sessions). In all but one study, the number of sessions followed the same schedule as standard care, although in keeping with the model, the group sessions were longer. The Barnes et al., (2017a, 2017b) study included a higher than standard number of antenatal sessions (14 vs 9) and also included 30 postnatal group sessions, compared to no or a single postnatal group session in the other studies. Target groups in the included studies ranged from young women and/or low-risk pregnancies to all pregnant women.

**Table 2:** Characteristics of included studies

**Study**	Country	Aim of economic analysis	Group care model (model description, number of sessions and duration, number of participants, provider)	Individual care model (number of visits and duration, provider)	Target group (type of risk for pregnancy, population, age group)
Ickovics et al., 2007	USA	Examine differences in reproductive health outcomes, psychological outcomes and health care costs between GANC and IANC	Centering Pregnancy, 10 structured sessions of 2 h, with 8 women, led by midwife or obstetrician	Unknown number of visits of 10—15 min to midwife or obstetrician	Low to moderate risk, young women, 14—25 years
Mooney et al., 2008	USA	Analyse cost of GANC compared to IANC	Centering Pregnancy, 10 group visits of 2.5 h and 2 return visits of 0.5 h, with unknown group size, led by midwife and physician	1 initial visit of 1 h and 12 return visits of 0.5 h to midwife or physician	Any risk, general population of women, no age range provided
Gareau et al., 2016	USA	Compare cost savings of prevention of adverse birth outcomes by GANC compared to IANC with cost of providing GANC	Centering Pregnancy, 10 group sessions of 2 h, with 8—12 women, provider not specified	(not reported)	Low risk, first-time mothers, 16—48 years
Rowley et al., 2016	USA	Compare cost and revenues for GANC and IANC	Centering Pregnancy (slightly modified), 4 individual visits of 40 min in last month, 10 prenatal and 1 postpartum group sessions of 2 h, with 8—12 women, led by nurse practitioner and 2 registered nurses	Intake visit of 1 h and 13 visits of 40 min to registered nurse or nurse practitioner	Any risk, general population of women, no age range provided
Barnes et al., 2017a, 2017b	UK	Assess cost-effectiveness of GANC&GPNC compared to IANC&IPNC	Group Family Nurse Partnership, 14 prenatal and 30 postnatal group meetings of 2 h, no group size specified, led by 2 experienced family nurses, one of whom had notified their intention to practice as a midwife (not in regular service)	Unknown number of visits with unknown duration with midwife or doctor	Any risk, vulnerable population, age below 20 years with one or more previous live births, or aged 20—24 at with no previous live births and with low educational qualifications
Crockett et al., 2017	USA	Comparing investment in GANC with net savings in NICU costs for GANC compared to IANC	Centering Pregnancy, ≤ 10 sessions of 1.5 h, with 8—12 women, led by nurse practitioners or certified nurse midwives	10—15 visits of 10—15 min, provider not specified	Exclusion of pregestational diabetes, hypertension, multiple gestation, high BMI, general population of women, mean age 24.5 years (SD 4.35)
Harsha Bangura et al., 2020	Nepal	Assess cost of implementation and provision of GANC	Centering Pregnancy adapted for low-resource setting, 6 sessions with unknown duration, about 8 women, led by nurse-midwife and community health worker	(not applicable)	Any risk, general population of women, no age range provided
McKinnon et al., 2020	Senegal	Estimate the fixed cost to introduce GANC	G-ANC, 4 sessions of 2 h, with 8—12 women, led by midwife or nurse and one matrone	4 visits with unknown duration to midwives or nurses	Any risk, general population of women, ≥ 15 years of age
Jans et al., 2023	Netherlands	Assess long-term cost–benefit of GANC compared to IANC	Centering-based, 8—10 sessions of 90 to 120 min, no group size specified, led by midwife or obstetrician and a co-facilitator(e.g. maternity care assistant)	A booking visit of 40 min, 12 individual visits of 17 min and 1 6-week postnatal visit of 27 min with midwife	Low risk, general population of women, no age range provided

*GANC* group antenatal care, *IANC* individual antenatal care, *GPNC* group postnatal care, *IPNC* individual postnatal care

The main appraisal issues from an economic perspective arose from incomplete descriptions of the competing (standard) forms of ANC provided as well as shortcomings in inclusion, identification, measurement and valuation of the costs (see Table 3). The concerns about the costs relate to the completeness (short follow-up), the assumptions made and how they are measured. Studies comparing GANC and IANC used randomised controlled designs (Barnes et al., 2017a, 2017b; Ickovics et al., 2007; Jans et al., 2023; McKinnon et al., 2020), a cohort or case–control design with propensity score matching (Crockett et al., 2017; Gareau et al., 2016) or a financial-economic model (Mooney et al., 2008; Rowley et al., 2016).

**Table 3:**
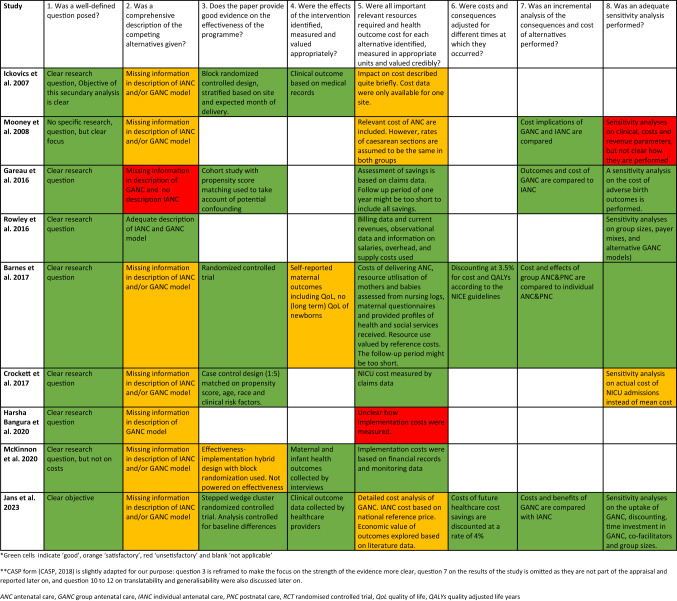
Critical appraisal* of included studies using the CASP economic evaluations checklist**

Most studies compared GANC and IANC in terms of costs and/or possible savings (see Table 4). However, the two studies from LMIC settings looked at the costs of implementing and providing GANC only (Harsha Bangura et al., 2020; McKinnon et al., 2020). Two other studies looked at the cost of providing GANC and compared it with savings in their local situation in the cost of NICU admissions (Crockett et al., 2017) or the cost of adverse birth outcomes for mother and child in the first year after birth (Gareau et al., 2016), both of which showed net savings for GANC.

**Table 4:** Economic aspects and results

**Study**	Type of cost data	Cost categories included	Perspective	Currency & year	Results
Ickovics et al., 2007	actual costs	• cost of GANC • cost of IANC • cost of delivery	hospital	US dollar 2001—2004	GANC: prenatal care $4,149 and delivery costs $3,433 IANC: prenatal care $4,091 and delivery costs $3,417
Mooney et al., 2008	billing data and hospital tax forms, cost reports	• cost of GANC • cost of IANC	hospital	US dollar 2005—2006	Financial breakeven point at 305 deliveries per year
Gareau et al., 2016	actual payments from one year before delivery for pregnant women and one year from delivery for mother and child	• cost of GANC • cost of adverse birth outcomes in 1 st year	hospital	US dollar 2009—2013	Savings for 1262 pregnant women are $3,989,214. Investment was $1,700,000 resulting in return on investment $2,289,214
Rowley et al., 2016	billing data, time observations, salary data and cost prices	• cost of GANC • cost of IANC	hospital	US dollar 2008—2013	Expenses are $746 per pregnancy for traditional care and $1184 for GANC, while revenues are $990 for traditional care and $1081 for GANC. Cost of group care using the CenteringPregnancy model are assessed at $684 per pregnancy
Barnes et al., 2017a, 2017b	activity logs of health practitioners, maternal questionnaires, profiles of hospital, community health and social services, unit costs from national sources	• cost of GANC • cost of IANC • cost of health & social services of mothers and babies in 1 st year after birth • costs of legal services and costs borne by trial participants or family members and friends	health- and social care	British pound 2015—2016	GANC: mean cost £8179 (pregnancy & 1 st year after birth) and mean effects 0.92 QALY IANC: mean cost £6107 (pregnancy & 1 st year after birth) and mean effects 0.93 QALY Probability that GANC is cost-effective did not exceed 3%
Crockett et al., 2017	claims data	• incentive payment GANC • cost of NICU admissions	hospital	US dollar 2013	Investing in CenteringPregnancy for 85 patients ($14,875) yielded a net savings for the MCO of $67,293 in NICU costs
Harsha Bangura et al., 2020	(not reported)	• cost of training • cost of GANC	healthcare	Nepali Rupees (US dollar) 2015—2016	Annual per capita cost $0.50, initial 3-day training and 2-day retraining of supervisors, CHWs, and nurse-midwives 92,750 Nepali Rupees ($861). Cost of intervention (CHW time, nurse supervisor time, portable ultrasounds, and lab supplies) are 4000 Nepali Rupees ($37) per woman completing four visits
McKinnon et al., 2020	financial records and monitoring data	• cost of training • cost of materials • material costs GANC	healthcare	West African franc (US dollar) 2017—2018	Fixed costs including materials and training are estimated at 210,000 FCFA ($357). Recurrent operational costs (mobile phone credit and refreshments provided to women during GANC sessions) are estimated at 48,000 FCFA ($81) for the four GANC sessions per group
Jans et al., 2023	maternity care tariff, gross salaries, actual costs, long-term healthcare cost-savings from literature	• cost of training • cost of GANC • cost of IANC • lifetime healthcare costs	healthcare	Euro 2019	GANC comes at a differential cost of €45 extra per person when compared to IANC. However, projected healthcare cost-savings of €112 related to increased breastfeeding rates, reduced prevalence of pregnancy induced hypertension and less postpartum smoking, lead to an average net cost-savings of €67 per GANC participant

*GANC* group antenatal care, *IANC* individual antenatal care, *GPNC,* group postnatal care, *IPNC* individual postnatal care

Mooney et al. (2008) and Rowley et al. (2016) compared the costs and revenues of GANC and IANC. Mooney et al. (2008) studied the financial impact of GANC in low obstetric volume settings and found that for 305 deliveries or more in a small, rural hospital, the costs will be lower than the revenues, assuming that 50% of the patients will choose group care. Rowley et al. (2016) created a financial model for GANC for use in an undersaved practice and reported higher costs than revenues for GANC while lower costs than revenues for IANC for their current situation. However, using larger group sizes or different staffing models were found to lead to financial sustainability of possibly a net income generator.

The other articles compared the costs of GANC and IANC also included the costs of childbirth (Ickovics et al., 2007) and the costs of providing the care for mothers and babies in the first year of the newborn (Barnes et al., 2017a, 2017b) or even the modelled lifetime health costs of the mother and child (Jans et al., 2023). Studies that only considered short-term costs (Ickovics et al., 2007, Rowley et al., 2016 and Barnes et al., 2017a, 2017b) showed more favourable results for IANC, while Jans et al., 2023, using a lifelong perspective, reported slightly lower healthcare costs for GANC.

A hospital perspective was most often used to assess whether the hospital's revenues exceeded the actual costs of providing group care (Ickovics et al., 2007; Mooney et al., 2008; Rowley et al., 2016) or to determine whether the additional actual costs (Gareau et al., 2016) or incentive payments (Crockett et al., 2017) of group care were covered by future savings in respectively healthcare costs in the first year or averted NICU admissions. The remaining studies took a broader perspective, not specifically related to a hospital but more generally on healthcare costs (Harsha Bangura et al., 2020; Jans et al., 2023; McKinnon et al., 2020) or costs of health and social care (Barnes et al., 2017a, 2017b) at country level.

Some of the studies carried out scenario and/or sensitivity analyses to explore different group care scenarios (Mooney et al., 2008; Rowley et al. 2015, Barnes et al., 2017a, 2017b; Jans et al., 2023). Scenarios differed concerning group size, number of sessions, staffing mix, payor mix, perspective, and uptake of GANC. Overall, the scenarios led to the expected results: small group sizes increase costs per pregnancy (Jans et al., 2023; Mooney et al., 2008; Rowley et al., 2016), more group sessions lead to higher costs (Rowley et al., 2016), higher attendance at group sessions leads to lower average costs (Barnes et al., 2017a, 2017b*)*, shifting care from higher cost providers to lower cost providers reduce costs (Jans et al., 2023; Mooney et al., 2008), higher uptake of GANC leads to higher costs (Jans et al., 2023), and in the study of Barnes et al., (2017a, 2017b*a,b*) broadening from a health and social care perspective to a societal perspective—including costs incurred by all sectors of the economy and by families and informal carers in a sensitivity analysis—has little effect on outcomes.

Table 5 summarises the different cost categories of GANC and IANC and how they compare as described in the included studies. Due to the different cost components and denominators (per woman, per practice/health post/health system) included in the different studies, results are difficult to compare. However, implementation costs were relatively low in the LMIC settings even after correcting for purchasing power differences, $1499 per health post (McKinnon et al., 2020) and $10,652 for 6 clusters of 2000 women (Harsha Bangura et al., 2020), respectively, and range from $8808 per practice in the Netherlands (Jans et al., 2023) to $2.0 million over 4 years in a US health system with 3000 deliveries per year, equating to $683 per person (Gareau et al., 2016). Costs of providing GANC showed the same picture, ranging from $34 per woman ($343 per group with an average of 10 women in McKinnon et al., 2020) and $459 per woman (Harsha Bangura et al., 2020) in the LMIC settings to $6111 per woman in one of the US settings (Ickovics et al., 2007). Studies comparing group care with individual care, ranged from GANC being cost saving with a reduction of $3818 in delivery and other healthcare costs in the first year after birth per woman (Gareau et al., 2016) to additional costs of $1001 per woman for delivery and other healthcare costs in the first year after birth for a combined GANC&GPNC programme which also included a much higher number of visits than in standard care (Barnes et al., 2017a, 2017b).

**Table 5:** Cost (components) of GANC and IANC, and their difference in the original currency and year (**US dollar, price level 2021, healthcare PPP adjusted**^*§*^***)***

Study	Currency & year	Cost of implementation				Cost of GANC		Cost of IANC	Potential healthcare savings of GANC^!^	
		Cost of training	Cost of equipment	Cost of materials	Cost of adapting venue	Personnel cost	Other cost		Savings in delivery costs (including NICU)	Savings in other healthcare cost
Ickovics et al., 2007	US dollar 2001—2004					$4149 **($6111)** per woman		$4091 **($6026)** per woman	-$16 **(-$24)** per woman	
Mooney et al., 2008	US dollar 2005—2006									
Gareau et al., 2016	US dollar 2009—2013	$1.7 **($2.0)** million over 4 years in a health system with 3000 deliveries per year*							$4.0 **($4.8)** million for 1262 women	
Rowley et al., 2016	US dollar 2008—2013					$778‡ **($938)** per woman	$406 **($489)** ‡ per woman	$746 **($899)**		
Barnes et al., 2017a, 2017b	British pound 2015—2016					£2036 **($3969)** (including GPNC) per woman†			-£51 **(-$99)** per woman	-£684 **(-$1333)** per woman
Crockett et al., 2017	US dollar 2013	$14,875**($17,302)** for 85 patients^¶^							$82,168 **($95,576)** for 85 patients	
Harsha Bangura et al., 2020	Nepali Rupees (US dollar) 2015—2016	92,750 NRS **($10,652)** for supervisors, child health workers and nurse-midwives from 6 clusters of 2000 people				4000 NRS **($459)** per woman				
McKinnon et al., 2020	West African franc (US dollar) 2017—2018	210,000 FCFA **($1499)** per health post					48,000 FCFA **($343)** per group			
Jans et al., 2023	Euro 2019	€4847.50 **($8808)** per practice	€150 **($273)** per practice	€250 **($454)** per practice	€250 **($454)** per practice	€625 **($1135)** per woman	€17 **($32)** per woman	€596 **($1083)** per woman		€112 **($204)** per woman

^§^Cost in US dollars, at 2021 price levels, are shown in bold. These figures are based on healthcare specific purchasing power parities (PPP). PPP are measures of the price of specific (healthcare) goods in different countries and are used to compare the absolute purchasing power of the countries' currencies

! Negative numbers means additional costs for GANC

^*^Including incentivized payments to obstetric providers adopting CenteringPregnancy

^‡^For 9 women per group

^†^Including training activities

^¶^Incentive payment for additional cost of GANC including supplies, administrative time, lost provider productivity, ongoing training and program certification

*GANC* group antenatal care, *IANC* individual antenatal care, *PPP* purchasing power parity, *GPNC* group postnatal care

## Discussion

A limited number of studies were found that addressed the costs and/or benefits of GANC. Studies also differ in the type of economic evaluation, with six partial evaluations, i.e. considering only GANC without a comparator (Harsha Bangura et al., 2020; McKinnon et al., 2020) or considering only costs and not health outcomes (Crockett et al., 2017; Ickovics et al., 2007; Mooney et al., 2008; Rowley et al., 2016), or considering only some cost factors and three full economic evaluations explicitly comparing the costs and outcomes of GANC with a comparator (Barnes et al., 2017a, 2017b; Gareau et al., 2016; Jans et al., 2023).

Although partial evaluations can provide useful information, they cannot alone guide decision-making, as simply knowing the cost difference of an intervention compared to usual care or cost and outcomes without a comparator does not indicate the value for money of an intervention (Turner et al., 2021). Moreover, the full economic evaluations identified vary considerably, for example, in terms of the perspective used and the follow-up period. The perspective refers to which costs are included in the evaluation and varied from a hospital perspective (including only hospital costs) (Gareau et al., 2016), health care perspective (including only health care costs) (Jans et al., 2023) to a broader perspective including social costs and even a societal perspective (Barnes et al., 2017a, 2017b). The follow-up period varied from costs only during pregnancy and the first year (Barnes et al., 2017a, 2017b; Gareau et al., 2016) to modelled lifetime costs (Jans et al., 2023). As some of the health effects and savings of GANC compared to IANC may occur later in the life of women and infants due to improved outcomes with GANC, limiting the follow-up period may lead to underestimation of potential cost savings, which would offset the typically somewhat higher costs per antenatal visit per woman for GANC.

This diversity in GANC's economic evaluations to date prevents meaningful comparisons of their results and, more importantly, evidence for widespread implementation. Therefore, more full economic evaluations are needed on GANC than IANC.

Conducting full economic evaluations of GANC versus IANC is not straightforward. For full economic evaluations of GANC compared to IANC, short-term data from trials or well-controlled cohort studies and lifetime data on the differences in costs and outcomes of GANC and IANC are needed. Differences in costs and outcomes are preferably measured in pragmatic randomised controlled trials. Although Barnes et al., (2017a, 2017b) managed to conduct a randomised controlled trial, it is not easy to organise GANC and IANC interventions in parallel groups due to the nature of GANC for which a group of women/parents should be enrolled. This problem is solved in some of the other studies by using more feasible designs, such as a block randomised controlled, and stepped-wedge cluster trial design (Ickovics et al., 2007; Jans et al., 2023; McKinnon et al., 2020) or cohort studies and case–control studies matched on propensity scores (Crockett et al., 2017; Gareau et al., 2016), but at the cost of a lower level of evidence. In addition, all the empirical studies included had limited follow-up. In contrast, the time horizon for an economic evaluation should cover the period over which the costs and/or outcomes of the alternative interventions being compared may differ (Drummond et al., 2015), which in this situation is the lifetime of mother and child as public health benefits such as increased breastfeeding or reduced preterm birth have been identified in trials. Decision models are needed to bridge the gap between what has been observed in empirical studies and what would be expected in terms of costs and effects over a long time horizon. Jans et al (2023) made a first attempt to model the long-term effects by including the long-term benefits of the intermediate outcomes on reduced pregnancy-induced hypertension-related cardiovascular disease, reduced passive smoking in children and increased breastfeeding initiation rates with GANC on maternal and child health. More research is needed on translating intermediate outcomes of empirical studies on GANC to lifetime outcomes.

Another important feature of the studies included in this review is that most were from the pilot or newly implemented GANC models, so the costs and performance may not be representative of, and presumably less favourable than, a more embedded and scaled-up model.

We did not include conference abstracts because of insufficient detail on methods and results (Nguyen et al. 2014, Meadows et al. 2019, Mazzoni et al. 2015), although they all point to savings due to improved birth procedures and outcomes.

### Strengths and Limitations of Review

This is the first review of the economics of GANC. We used a rigorous and systematic methodology to identify and evaluate the studies included aligning to methodological guidelines of review (Li et al., 2019). A full overview of the available information is provided.

The limitations of this review are that we had a limited number of studies from which to draw conclusions. In addition, the variability of the design and other study characteristics, such as the cost categories included and the follow-up period, made it impossible to reach an evidence-based synthesis and definitive (guideline) statements. However, no matter how uniformly cost studies are conducted, the understanding of the cost implications of each study will depend largely on the context of each country. Health system factors at the macro, meso and micro levels will have a strong influence on cost implications. In some countries, GANC may increase costs, while the same GANC model may reduce costs in other countries.

### Implications

Although the current evidence base on the costs of GANC is limited, the available findings suggest that any higher costs per visit may be offset by potential improvements in short- or long-term maternal and child outcomes. This raises the possibility that GANC could offer value for money. However, the absence of robust economic evaluations makes it difficult for policymakers and health systems to assess its true cost implications.

Heterogeneity in study designs, cost components and outcome measures also limits comparability across studies, highlighting the need for standardised economic reporting. Future research should incorporate full economic evaluations alongside clinical and wellbeing outcomes and include both direct and indirect costs. Follow-up should be extended, for example by modelling, to capture lifetime health trajectories and costs. Strengthening this evidence base is essential for informing implementation decisions, guiding resource allocation and determining whether GANC could be a sustainable and equitable alternative to IANC within different health systems.

## Conclusions

Evidence on the costs of GANC is sparse. Based on this review and a narrative summary, there is some indication that costs per visit per woman are likely higher but may be offset by improvement in short or long-term outcomes. However, the confidence level in the evidence is low and do not allow firm conclusions about the economic implications. High-quality studies that include comprehensive economic evaluations are needed to clarify the (long term) cost and outcome differences between GANC and IANC.

## Supplementary Information

Below is the link to the electronic supplementary material.

Supplementary file1 (PDF 112 KB)
