# Supplementary material for: The Economics of Group Antenatal Care: A Systematic Review and Narrative Analysis
Source: Matern Child Health J. 2026 May 13;30(6):820–30. doi: 10.1007/s10995-026-04276-x (PMC13275586; doi:10.1007/s10995-026-04276-x)
Supplement: Supplementary file 1 — Supplementary file1 (PDF 112 KB) [file 10995_2026_4276_MOESM1_ESM.pdf]

SUPPLEMENTARY TABLE Search strategy

PUBMED

(“Cost-Benefit Analysis"[Mesh] OR "Health Care Costs"[Mesh] OR "economic evaluations"[Title/Abstract] OR "economic evaluation"[Title/Abstract] OR "Cost-Benefit"[Title/Abstract] OR "Benefits and Costs"[Title/Abstract] OR "Cost Benefit"[Title/Abstract] OR "Cost Effectiveness"[Title/Abstract] OR "Cost-Effectiveness"[Title/Abstract] OR "Cost Effective"[Title/Abstract] OR "Cost Utility"[Title/Abstract] OR "Cost-Utility"[Title/Abstract] OR "Costs and Benefits"[Title/Abstract] OR "Marginal Analysis"[Title/Abstract] OR "Cost savings" [Title/Abstract] OR "Cost Analysis" [Title/Abstract] OR "Financial" [Title/Abstract] OR "Cost" [Title/Abstract] OR "Economic" [Title/Abstract])

AND

("circle class" [Title/Abstract:~1] OR "circle classes" [Title/Abstract:~1] OR "circle screening" [Title/Abstract:~1] OR "circle assessment" [Title/Abstract:~1] OR "circle assessments" [Title/Abstract:~1] OR "circle checkup" [Title/Abstract:~1] OR "circle checkups" [Title/Abstract:~1] OR "circle check-up" [Title/Abstract:~1] OR "circle check-ups" [Title/Abstract:~1] OR "circle check up" [Title/Abstract:~1] OR "circle check ups" [Title/Abstract:~1] OR "group education" [Title/Abstract:~1] OR "group class" [Title/Abstract:~1] OR "group classes" [Title/Abstract:~1] OR "group care" [Title/Abstract:~1] OR "group screening" [Title/Abstract:~1] OR "group assessment" [Title/Abstract:~1] OR "group checkup" [Title/Abstract:~1] OR "group checkups" [Title/Abstract:~1] OR "group check-up" [Title/Abstract:~1] OR "group check-ups" [Title/Abstract:~1] OR "group check up" [Title/Abstract:~1] OR "group check ups" [Title/Abstract:~1] OR "Group Family Nurse Partnership" [Title/Abstract] OR "gFNP" [Title/Abstract] OR "centering" [Title/Abstract])

AND

("Prenatal care"[Mesh] OR "Postnatal care"[Mesh] OR "pre-natal" [Title/Abstract] OR "prenatal" [Title/Abstract] OR "peri-natal" [Title/Abstract] OR "perinatal” [Title/Abstract] OR "ante-natal" [Title/Abstract] OR "antenatal" [Title/Abstract] OR "childbirth" [Title/Abstract] OR "parturition" [Title/Abstract] OR "obstetr*" [Title/Abstract] OR "neonatal" [Title/Abstract] OR "neo-natal" [Title/Abstract] OR "midwife" [Title/Abstract] OR "midwives" [Title/Abstract] OR "matern*" [Title/Abstract] OR "antepartum" [Title/Abstract] OR "ante-partum" [Title/Abstract] OR "peripartum" [Title/Abstract] OR “peri-partum" [Title/Abstract] OR "pregnancy" [Title/Abstract])

EMBASE

1 ((circle adj2 class) or (circle adj2 classes) or (circle adj2 screening) or (circle adj2 assessment*) or (circle adj2 checkup*) or (group adj2 education) or (group adj2 class) or (group adj2 classes) or (group adj2 care) or (group adj2 screening) or (group adj2 assessment) or (group adj2 check up) or "Group Family Nurse Partnership" or "Gfnp" or "centering").ti,ab,kw.

2 cost utility analysis/

3 (cost* adj2 utilit*).ti,ab,kw.

4 (cost* adj2 (effective* or assess* or evaluat* or analys* or model* or benefit* or threshold* or quality or expens* or saving* or reduc*)).ti,ab,kw.

5 (economic* adj2 (evaluat* or assess* or analys* or model* or outcome* or benefit* or threshold* or expens* or saving* or reduc*)).ti,ab,kw.

6 (QALY* or (incremental* adj2 cost*) or ICER or utilities or markov*).ti,ab,kw.

7 (dollar* or USD or cents or pound or pounds or GBP or sterling* or pence or euro or euros or yen or JPY).ti,ab,kw.

8 2 or 3 or 4 or 5 or 6 or 7

9 prenatal care/

10 childbirth/

11 obstetrics/

12 nurse midwife/ or midwife/

13 maternal care/ or maternal child health care/

14 pregnancy/

15 ("pre-natal" or "prenatal" or "peri-natal" or "perinatal" or "ante-natal" or "antenatal" or "childbirth" or "parturition" or "obstetr*" or "neonatal" or "neo-natal" or "midwife" or "midwives" or "matern*" or "antepartum" or "ante-partum" or "peripartum" or "peri-partum" or "pregnancy").ti,ab,kw.

16 9 or 10 or 11 or 12 or 13 or 14 or 15

17 1 and 8 and 16

Ovid Emcare

1 ((circle adj2 class) or (circle adj2 classes) or (circle adj2 screening) or (circle adj2 assessment*) or (circle adj2 checkup*) or (group adj2 education) or (group adj2 class) or (group adj2 classes) or (group adj2 care) or (group adj2 screening) or (group adj2 assessment) or (group adj2 check up) or "Group Family Nurse Partnership" or "Gfnp" or "centering").ti,ab,kw.

2 cost utility analysis/

3 (cost* adj2 utilit*).ti,ab,kw.

4 (cost* adj2 (effective* or assess* or evaluat* or analys* or model* or benefit* or threshold* or quality or expens* or saving* or reduc*)).ti,ab,kw.

5 (economic* adj2 (evaluat* or assess* or analys* or model* or outcome* or benefit* or threshold* or expens* or saving* or reduc*)).ti,ab,kw.

6 (QALY* or (incremental* adj2 cost*) or ICER or utilities or markov*).ti,ab,kw.

7 (dollar* or USD or cents or pound or pounds or GBP or sterling* or pence or euro or euros or yen or JPY).ti,ab,kw.

8 2 or 3 or 4 or 5 or 6 or 7

9 prenatal care/

10 childbirth/

11 obstetrics/

12 nurse midwife/ or midwife/

13 maternal care/ or maternal child health care/

14 pregnancy/

15 ("pre-natal" or "prenatal" or "peri-natal" or "perinatal" or "ante-natal" or "antenatal" or "childbirth" or "parturition" or "obstetr*" or "neonatal" or "neo-natal" or "midwife" or "midwives" or "matern*" or "antepartum" or "ante-partum" or "peripartum" or "peri-partum" or "pregnancy").ti,ab,kw.

16 9 or 10 or 11 or 12 or 13 or 14 or 15

17 1 and 8 and 16
